# Alpha-Lipoic Acid Mediates Clearance of Iron Accumulation by Regulating Iron Metabolism in a Parkinson’s Disease Model Induced by 6-OHDA

**DOI:** 10.3389/fnins.2020.00612

**Published:** 2020-06-25

**Authors:** Shengyan Tai, Qian Zheng, Suzhen Zhai, Ting Cai, Li Xu, Lizhu Yang, Ling Jiao, Chunlin Zhang

**Affiliations:** ^1^Department of Biology, College of Basic Medical, Guizhou Medical University, Guiyang, China; ^2^Department of Neurology, The Affiliated Hospital of Guizhou Medical University, Guiyang, China

**Keywords:** alpha-lipoic acid, oxidative stress, chelation iron, Parkinson’s disease, iron homeostasis

## Abstract

The disruption of neuronal iron homeostasis and oxidative stress are related to the pathogenesis of Parkinson’s disease (PD). Alpha-lipoic acid (ALA) is a naturally occurring enzyme cofactor with antioxidant and iron chelator properties and has many known effects. ALA has neuroprotective effects on PD. However, its underlying mechanism remains unclear. In the present study, we established PD models induced by 6-hydroxydopamine (6-OHDA) to explore the neuroprotective ability of ALA and its underlying mechanism *in vivo* and *in vitro*. Our results showed that ALA could provide significant protection from 6-OHDA-induced cell damage *in vitro* by decreasing the levels of intracellular reactive oxygen species and iron. ALA significantly promoted the survival of the dopaminergic neuron in the 6-OHDA-induced PD rat model and remarkably ameliorated motor deficits by dramatically inhibiting the decrease in tyrosine hydroxylase expression and superoxide dismutase activity in the substantia nigra. Interestingly, ALA attenuated 6-OHDA-induced iron accumulation both *in vivo* and *in vitro* by antagonizing the 6-OHDA-induced upregulation of iron regulatory protein 2 and divalent metal transporter 1. These results indicated that the neuroprotective mechanism of ALA against neurological injury induced by 6-OHDA may be related to the regulation of iron homeostasis and reduced oxidative stress levels. Therefore, ALA may provide neuroprotective therapy for PD and other diseases related to iron metabolism disorder.

## Introduction

Parkinson’s disease (PD) is the second most common neurodegenerative disorder with a significant prevalence in elderly people. It is associated with the loss of striatal dopamine (DA) and degeneration of dopaminergic neurons in the substantia nigra (SN) (Angelini, 2017). Although several pathogenic mechanisms have been proposed to elucidate the nosogenesis of PD, such as genetics, environmental factors, oxidative stress, apoptosis, metal ion aggregation, and abnormal protein aggregation, the precise pathogenic mechanisms remain unclear (Dauer and Przedborski, 2003; Phani et al., 2012; Zhong et al., 2017).

Iron is required as a cofactor in metabolic processes in the body and specifically in high oxygen-consuming tissues, such as the central nervous system. However, in excess, iron is potentially cytotoxic because it can generate reactive oxygen species (ROS) by participating in redox reaction and then cell death. Abnormally high levels of iron in the brain have been demonstrated in a number of neurodegenerative disorders, such as PD and Alzheimer’s disease (AD) (Ong and Farooqui, 2005; Berg et al., 2006). Oxidative stress, resulting from increased brain iron levels, and possibly also from defects in antioxidant defense mechanisms, is widely believed to be associated with neuronal death in these disorders (Berg et al., 2006). Iron metabolism dysfunction plays a key role in PD (Ward et al., 2014; Jiang et al., 2017). Enhanced iron level has been observed in the SN of PD patients and animal models, and iron accumulation has also been shown in an *in vitro* model (Sofic et al., 1991; Wang Y. Q. et al., 2015). The altered expression of iron-related proteins in the SN may be responsible for the nigral iron accumulation in PD. Brain iron metabolism involves several proteins, such as divalent metal transporter 1 (DMT1) and iron regulatory proteins (IRPs). Two cytosolic iron sensors, namely, IRP1 and 2, regulate iron metabolism posttranscriptionally. Gene knockout studies on mice have shown that IRP2 is particularly important in the iron homeostasis of central nervous cells (Ghosh et al., 2015). IRP2 is an RNA-binding protein that can regulate intracellular iron homeostasis by binding iron responsive elements (IREs) of DMT1. Iron accumulation in PD is caused by IRP2, which increases iron uptake by regulating DMT1 (Jiang et al., 2017).

Alpha-lipoic acid (ALA) is a naturally occurring enzyme cofactor with antioxidant and iron chelator properties and has been used as a therapeutic agent for many diseases, such as cardiovascular diseases, hypertension, and diabetes (Rochette et al., 2013; Park et al., 2014). ALA also provides neuroprotection against PD because it can penetrate the blood–brain barrier. However, the associated mechanism remains unclear (Jalali-Nadoushan and Roghani, 2013; Zhao et al., 2017; Zhou et al., 2018a). ALA can reduce iron in cells and tissues (Goralska et al., 2003; Lal et al., 2008; Wang Y. et al., 2015; Chen et al., 2017). So far, few studies have reported about the effects of ALA on iron accumulation and its underlying mechanism in PD.

In the current study, we found that ALA attenuated 6-hydroxydopamine (6-OHDA)-induced iron accumulation both *in vivo* and *in vitro* by antagonizing the 6-OHDA-induced upregulation of IRP2 and DMT1. Therefore, we present a possible neuroprotective mechanism of ALA against neurological injury in a PD model induced by 6-OHDA by regulating iron metabolism to scavenger iron accumulation.

## Materials and Methods

### Chemicals

Alpha-lipoic acid, 6-OHDA, desipramine hydrochloride, and apomorphine were purchased from Sigma (United States). Prussian Blue Iron Stain Kit and 3-(4,5-dimethylthiazol-2-yl)-2,5-diphenyltetrazolium bromide (MTT) were purchased from Solarbio (Beijing, China). Tissue iron, total superoxide dismutase (SOD), and reduced glutathione (GSH) assay kits were purchased from Nanjing Jiancheng Bioengineering Institute (Nanjing, China). ROS assay kit was purchased from Beyotime (Shanghai, China). All other chemicals and reagents were of the highest grade available from local commercial sources.

### Antibodies

The following antibodies were used for immunoblotting: DMTI rabbit monoclonal antibody (Boster Biological Technology Ltd.), IRP2 rabbit polyclonal antibody (Santa Cruz Biotechnology), and β-actin rabbit monoclonal antibody (Sigma). The TH mouse monoclonal antibody (Boster Biological Technology Ltd.) was used for immunohistochemistry. All the secondary antibodies were purchased from Life Technologies. All primary antibodies were used at 1:1000 dilution for western blot analyses. All primary antibodies were diluted 1:100 for immunohistochemistry staining. The secondary antibodies for western blot analysis were diluted at 1:10,000. The secondary antibodies for immunohistochemistry staining were diluted at 1:200.

### Animals and Drug Treatments

Adult male Sprague–Dawley (SD) rats, weighing 250–300 g, were supplied by the Experimental Animal Centre of Guizhou Medical University. All animal experiments were performed in accordance with the National Institutes of Health Guide for the Care and Use of Laboratory Animals and approved by the Ethical Committee of the Guizhou Medical University.

Sprague–Dawley rats (*n* = 55; 7–8 weeks old; body weight 250–300 g) were maintained under standard laboratory conditions. Rats were randomly divided into the sham group (*n* = 15) and 6-OHDA lesion group (*n* = 40). 6-OHDA lesions were performed as previously described. In brief, 30 min before operations, the rats were pretreated with desipramine (25 mg/kg, i.p.), anesthetized with chloral hydrate (400 mg/kg, i.p.), and fixated in a stereotaxic frame. Ten micrograms of 6-OHDA (2 μL of 5 μg/μL solubilized in saline containing 0.2 mg/mL ascorbic acid to prevent oxidation) was injected into the right striatum of the 6-OHDA lesion group by using a 10-μL gauge Hamilton syringe at the two following coordinates: (a) A/P 1.5 mm, L/M 2.7 mm right, D/V 4.5 mm from Bregma and (b) A/P 0 mm, L/M 3.5 mm right, D/V 5 mm from Bregma. A total of 20 μg of 6-OHDA was injected at the two coordinates (10 μg for each site). 6-OHDA was delivered at 0.5 μL/min. The injection cannula (syringe) was retained in place for 10 min before being retracted slowly (1 mm/min). The sham group received an identical volume of vehicle (sterile water with 0.2 mg/mL L-ascorbic acid) similarly. Four weeks after the lesion was induced with 6-OHDA, the successfully established animal models (screening by behavior test) were randomly divided into the PD and treated groups. We selected 100 mg/kg of ALA as treatment concentration of our experiments based on some studies (de Araújo et al., 2013; Jalali-Nadoushan and Roghani, 2013; Zhou et al., 2018a). The treated group was administered with ALA (100 mg/kg) by i.p. injection consecutively for 14 days. The other group was concurrently administered an equivalent dose of sterile water.

### Apomorphine Rotation Test

The apomorphine-induced rotation test was performed at 4 and 6 weeks after surgery. The rats were placed in a cylindrical cage (240 mm in diameter, 300 mm height) and allowed to habituate for 15 min (Dabbeni-Sala et al., 2001). Apomorphine (2 mg/kg, s.c.) was subcutaneously administered to the 6-OHDA-treated rats. Approximately 10 min after administration, the rats were video recorded for 30 min. The number of apomorphine-induced rotations was counted.

### Cylinder Test

The cylinder test evaluated the motor deficits in the left forelimb by using vertical exploratory activity (Meredith and Kang, 2006). Rats with a unilateral 6-OHDA lesion would preferentially use the limb ipsilateral to the lesion. After 4 and 6 weeks of lesion induction, the rats were placed in an upright transparent cylinder (20 cm diameter, 30 cm height). The number of forelimb contacts on the cylinder wall with the left paw (contralateral lesion, CP), right paw (ipsilateral lesion, IP), and both paws (2P) was counted simultaneously during the final 5 min. The percentage of (CP + 2P) touches relative to the total number of touches (IP + CP + 2P = total) was calculated (Zhou et al., 2018a).

### Cell Culture and Drug Treatments

PC12 cells were obtained from the Conservation Genetics CAS Kunming Cell Bank. The cells were cultured in Dulbecco’s modified Eagle’s medium (HyClone) supplemented with 10% fetal bovine serum (HyClone) and 50 U/mL each of penicillin and streptomycin (Invitrogen) in a humidified incubator with 5% CO2 at 37°C. For the experiments, cells were seeded in plates and grown to 70–80% confluency before treatment with ALA for 1 h. 6-OHDA was then added and treated for another 24 h, and the cells were harvested for experiments.

### Cell Viability Assay

Cell viability assay was performed using MTT that can be reduced to purple-colored formazan by intact cells. After various treatments, cell viability was assessed using the Cell Titer 96 Aqueous One Solution Cell Proliferation Assay in accordance with the manufacturer’s instructions. The absorbance was measured with a UV spectrophotometer at a wavelength of 490 nm. Results are presented as a percentage of the control.

### Immunohistochemistry

The brain tissue of rats was anesthetized and perfused with 0.9% saline, followed by 4% paraformaldehyde (PFA). Brain samples were immersed in 4% PFA overnight. The samples were rinsed in double-distilled water for 30 min and then placed in 70% alcohol overnight. The samples were dehydrated by graded ethanol and embedded with wax block. The brain tissues were sectioned on a slicer at a 30-μm section. The sections were immersed with 3% H_2_O_2_ for 10 min and blocked with 5% BSA for 20 min and then incubated with a primary antibody (anti-TH, 1:100) for 2 h and an appropriate anti-mouse secondary antibody for 1 h. Immunostaining was performed by a commercial kit (DAB stain kit) for 10 min. The samples were visualized under a fluorescence microscope. TH-positive dopaminergic neurons were counted manually at 3400 magnification, and the cell counts were averaged from three non-overlapped fields per slide of the SNpc region. All counts were performed blindly by a person unaware of the groupings of animals.

### Detection of SOD and GSH in SN

The rats were beheaded, and SN was removed on ice. The tissues were separated and weighed on an accurate electronic scale. The cold homogenate was then added into the tissues. After the homogenate reached 10%, the tissues were centrifuged at 4°C for 10 min. The supernatant was obtained. The SOD and GSH activities were determined by hydroxylamine and spectrophotometric methods, respectively. The analyses strictly complied with the instructions of the reagent kit.

### Measurement of Intracellular ROS

Intracellular ROS levels were estimated following treatment with various compounds by using 2′,7′-dichlorofluorescein diacetate (H2DCFDA) as a fluorescent probe. PC12 cells were exposed to ALA and 6-OHDA for 24 h, and the culture medium was replaced with fresh serum-free medium containing 20 μmol/L H2DCFDA. The DCF fluorescence intensity was determined using inversion fluorescence microscope and ImageJ software.

### Determination of Total Iron Content

The total iron content of PC12 cells and the SN of rats were determined using a tissue iron assay kit according to the manufacturer’s directions. The tissue iron assay kits provide a simple convenient means of measuring ferrous (Fe2+) and/or ferric (Fe3+) iron in biological samples. In the iron assay protocol, ferrous iron (Fe2+) reacts with Ferene S to produce a stable colored complex with absorbance at 593 nm. Ferric iron (Fe3+) can be reduced to form Fe2+, enabling the measurement of total iron (Fe2+ and Fe3+). The level of ferric iron (Fe3+) is calculated by subtracting ferrous iron from total iron. The absorbance was read at 520 nm by using an UV spectrophotometer.

### Perls’ Iron Histochemistry

Iron staining was performed by using Perls’ Stain Kit in accordance with the manufacturer’s directions. Sections were fixed in 4% formaldehyde for 5 min, washed in Milli-Q water for 30 s before staining, and incubated for 30 min in a freshly prepared solution of equal parts 2% HCl and 2% potassium ferrocyanide. After washing with PBS, sections were dehydrated, cleared to transparency, and sealed in graded ethanol. Cell count was analyzed for TH staining.

### Western Blot

PC12 cells and SN tissues were lysed with RIPA lysis buffer (50 mM Tris pH 7.4, 150 mM NaCl, 1% NP-40, 0.5% sodium deoxycholate, 0.1% SDS, and PMSF), and the sample was placed on ice for 40 min. The lysates were then centrifuged at 12,000 r/min for 30 min, and the supernatants were used for analysis. Protein concentrations were determined by using a BCA Protein Assay Kit (Beyotime). The supernatants were diluted to 40 μg/lane with a sample buffer and heated at 95°C for 5 min. The protein mixtures were loaded on a 12% SDS-PAGE gel and transferred to polyvinylidene difluoride membranes. The membranes were blocked with 5% non-fat milk in TBST for 1.5 h and then incubated overnight at 4°C with primary antibodies: anti-β-actin, anti-IRP2, and anti-DMT1. The blots were washed and incubated with anti-rabbit IgG for 1 h at room temperature. Cross-reactivity was visualized using ECL Western Blotting Detection Reagents, and all bands were visualized and analyzed by ImageJ.

### Statistical Analysis

Statistical analyses were performed using SPSS 19.0 software. The data were expressed as mean ± SD of three independent experiments. One-way ANOVA followed by Turkey’s test was used to compare the differences between means. A probability value of *P* < 0.05 was considered to be statistically significant.

## Results

### ALA Ameliorates Motor Behavior and Prevents DA Neuron Loss in SN of PD Rat Models

#### Apomorphine-Induced Rotation Behavior

To screen for PD model rats, we analyzed the rats by apomorphine-induced rotation test at 4 weeks after 6-OHDA injections. The successfully established animal models, with a rotation speed over 7 r/min, were randomly divided into PD model group (*n* = 15) and ALA treatment group (*n* = 15). Rats in the sham group had no rotation after apomorphine injections.

**FIGURE 1:**
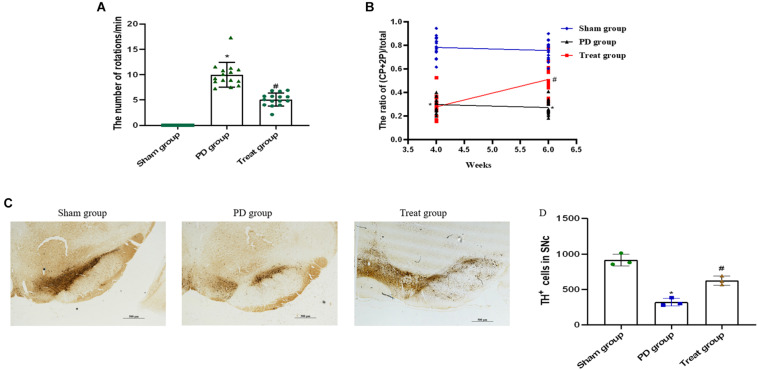
ALA ameliorates motor behavior and prevents DA neuron loss in the SN of PD rat models. ALA ameliorates apomorphine-induced rotation behavior **(A)** and fine motor activity in the 6-OHDA-induced rat model of PD **(B)**. Data were expressed as the means ± SD (*n* = 15/group) and analyzed by one-way ANOVA. The immunohistochemical staining of TH-positive neurons in the SN. Scale bar, 500 μm **(C)**. The number of TH-positive cells was averaged from three non-overlapped fields per slide of the SNpc region. **(D)** Quantification of TH-positive neurons in the SN. Data were presented as the mean ± SD (*n* = 3). Statistical significance: *P < 0.01 compared with the sham group; ^#^*P* < 0.01 compared with the PD group by ANOVA.

To evaluate the effect of ALA on motor coordination and balance, we analyzed each group of rats by apomorphine-induced rotation test at 6 weeks after 6-OHDA injections. The results showed that the number of body rotations (the direction opposite to the side of the lesion) in the PD group was increased drastically compared with the sham group (sham group, 0 ± 0; PD group, 9.98 ± 2.45; *P* = 0.0003). The number of rotations was significantly decreased after treatment with ALA (treat group, 5.09 ± 1.30, *P* = 0.001) (Figure 1A).

#### Cylinder Test

The cylinder test is sensitive to the degree of DA level in the SN and has been used as a tool for assessing fine motor activity in PD. It was performed 4 weeks and 6 weeks after 6-OHDA injections. The current results showed that the percentage of CP + 2P was significantly decreased in the PD group compared with the sham group at 4 weeks (sham group, 0.79 ± 0.09; PD group, 0.30 ± 0.06; *P* = 0.0001), and 6 weeks after 6-OHDA injections (sham group, 0.76 ± 0.08; PD group, 0.27 ± 0.06; *P* = 0.001). ALA treatment could evidently increase the percentage of CP + 2P compared with the PD group at 6 weeks after 6-OHDA injections (treat group, 0.51 ± 0.14, *P* = 0.0012). However, there was no significant difference in the treat group compared with the PD group at 4 weeks after 6-OHDA injections, because it is the second day of ALA treatment (treat group, 0.28 ± 0.10, *P* = 0.5210) (Figure 1B).

#### ALA Inhibited the Decrease in TH Expression in the SN of Rats

To observe the neuroprotective effect of ALA on DA neurons, we examined the number of TH-positive neurons in the SN. The results showed that the PD group exhibited a significant decrease in the number of TH-positive neurons (sham group, 916.67 ± 84.36; PD group, 321.00 ± 54.81; *P* = 0.0001), which could be inhibited by ALA treatment (treat group, 625.67 ± 63.45, *P* = 0.002) (Figures 1C,D).

### ALA Protects Against 6-OHDA-Induced Damage in PC12 Cells

**FIGURE 2:**
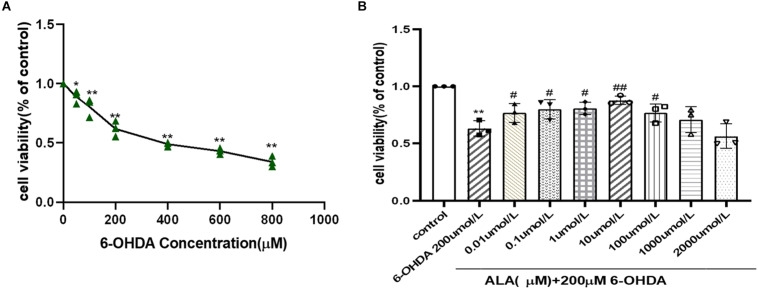
ALA protected against 6-OHDA-induced damage of PC12 cells. **(A)** PC12 cells were incubated with different concentrations of 6-OHDA for 24 h. **(B)** PC12 cells were pretreated with ALA for 1 h and then exposed to 6-OHDA for 24 h. Data were expressed as the means ± SD analyzed by one-way ANOVA. Statistical significance: *P < 0.05, **P < 0.01 vs. the control group; ^#^*P* < 0.05, ^##^*P* < 0.01 vs. the PD group.

PC12 cells were incubated with different concentrations (50–800 μM) of 6-OHDA, and cell viability was expressed as an MTT conversion rate. 6-OHDA induced cell death in a dose-dependent manner, as shown by survival rates of PC12 cells at 87 and 39% when treated with 50 and 800 μM 6-OHDA for 24 h, respectively. The PD cell model induced by 6-OHDA requires appropriate cell viability. The excessive cell damage will cause irreversible damage; however, insufficient cell damage will interfere the observation of drug treatment. The cell viability is about 60% when the concentration of 6-OHDA is 200 μmol/L (0.62 ± 0.07, *P* = 0.0001). Hence, 200 μmol/L of 6-OHDA was selected for the PD cell model concentration of subsequent experiments (Figure 2A). The cells were pretreated with different concentrations of ALA for 1 h and exposed to 200 μM 6-OHDA for an additional 24 h. The results showed that ALA clearly mitigated the 6-OHDA-induced decrease in cell viability, and 10 μM of ALA was the peak of its protective effect (10 μM, 0.88 ± 0.04; *P* = 0.0010) (Figure 2B). Therefore, 10 μM of ALA was selected for the treatment concentration of subsequent experiments.

### ALA Inhibits the Decrease in the Activity of SOD and GSH in the SN of a Rat Model of PD Induced by 6-OHDA

**FIGURE 3:**
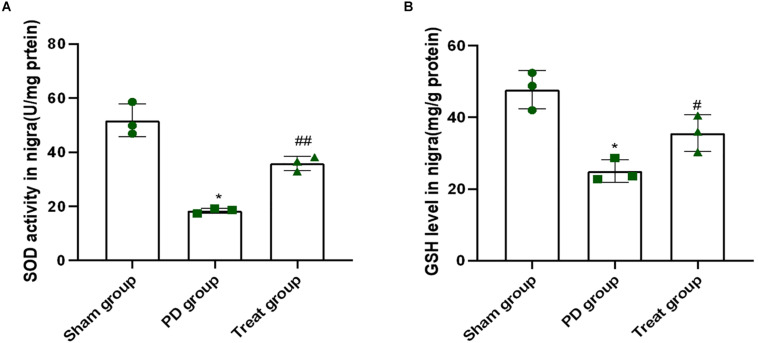
ALA inhibits the decrease in the activity of SOD and GSH in the SN of the rat model of PD induced by 6-OHDA. **(A)**. The activity of SOD in the SN was dramatically suppressed by 6-OHDA and was rescued by ALA. **(B)** The level of GSH in the SN was significantly suppressed by 6-OHDA and was rescued by ALA. Data were expressed as the means ± SD analyzed by one-way ANOVA (*n* = 3). Statistical significance: *P < 0.01 vs. sham group; ^#^*P* < 0.05, ^##^*P* < 0.01 vs. PD group.

Superoxide dismutase and reduced glutathione play important roles in clearing free radicals in the body, which are associated with many pathological processes, including PD. As shown in Figure 3, when rats were treated with 6-OHDA, the activity of SOD and contents of GSH in the SN was 18 U/mg protein and 25 mg/g protein, respectively (SOD: sham group, 51.77 ± 6.08; PD group, 18.41 ± 0.94; *P* = 0.0001. GSH: sham group, 47.74 ± 5.29; PD group, 25.03 ± 3.15; *P* = 0.0010). This finding indicated that 6-OHDA dramatically suppressed the activity of SOD and contents of GSH. However, the activity of SOD and GSH content was 36 U/mg protein and 36 mg/g protein, respectively, in rats after ALA treatment, which were higher than those of the PD model group (SOD: treat group, 35.90 ± 2.67, *P* = 0.0010, GSH: treat group, 35.63 ± 5.10, *P* = 0.0310) (Figures 3A,B). These results indicated that ALA significantly rescued the decrease in SOD activity and GSH contents induced by 6-OHDA.

### ALA Attenuates 6-OHDA-Induced Increase in ROS Levels of PC12 Cells

**FIGURE 4:**
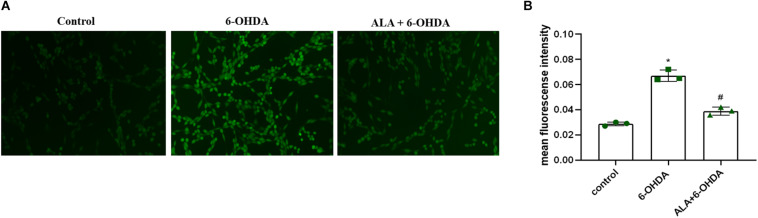
ALA attenuates 6-OHDA-induced increase in ROS levels of PC12 cells. **(A)** Corresponding DCF-fluorescent picture induced by 200 μM 6-OHDA and/or 10 μM ALA in PC12 cells. The brightened dot represents the fluorescence of ROS. **(B)** DCF fluorescence intensity induced by 200 μM 6-OHDA and/or 10 μM ALA in PC12 cells. Data were expressed as the means ± SD analyzed by one-way ANOVA (*n* = 3). Statistical significance: *P < 0.01 vs. control; ^#^*P* < 0.01 vs. 6-OHDA group.

To explore the effect of ALA on ROS levels, we exposed PC12 cells to 200 μM of 6-OHDA for 24 h. Results showed significantly increased fluorescence intensity compared with control (control, 0.028 ± 0.0020; 6-OHDA, 0.067 ± 0.044; *P* = 0.0001), which was eliminated dramatically by pretreating the cells with 10 μM of ALA (ALA + 6-OHDA, 0.039 ± 0.031; *P* = 0.0003) (Figures 4A,B).

### ALA Suppresses the 6-OHDA-Induced Excessive Iron Accumulation in PC12 Cells and SN of Rats

**FIGURE 5:**
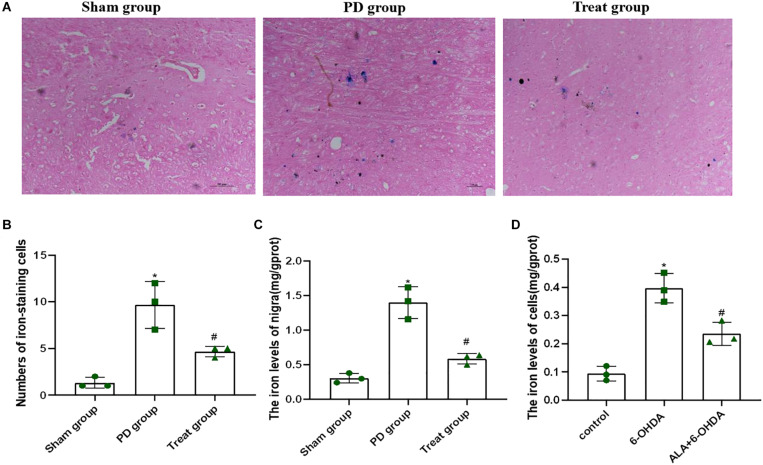
ALA suppresses the 6-OHDA-induced excessive iron accumulation in the SN of rats and PC12 cells. **(A)** Iron-staining cells in the SN of rats. **(B)** Corresponding numbers of iron-staining cells in the SN of rats. **(C)** The total iron content in the SN of rats was detected by using a tissue iron assay kit. **(D)** The total iron content of PC12 cells was detected by using a tissue iron assay kit. Data were expressed as the means ± SD analyzed by one-way ANOVA (*n* = 3). Statistical significance: *P < 0.01 vs. sham group or control; ^#^*P* < 0.01 vs. PD group or 6-OHDA.

Increased iron levels in the SN play an important role in the etiology of PD. To determine the effect of ALA on 6-OHDA-induced iron deposit in the SN of rats, we first detected the number of iron-staining cells in the SN with Perls’ iron histochemistry. As shown in Figures 5A,B, the number of iron-staining cells in the SN of the PD model group was significantly increased compared with that of the sham group (sham group, 1.33 ± 0.58; PD group, 9.67 ± 2.52; *P* = 0.0010). However, after ALA treatment, the number of iron-staining cells was significantly reduced (treat group, 4.67 ± 0.58; *P* = 0.0070). Furthermore, we also tested the total iron content in SN with tissue iron assay kit, showing that the total iron content of the PD model group was significantly increased compared with that of the sham group (sham group, 0.31 ± 0.07, PD group, 1.401 ± 0.23; *P* = 0.0001), which could be inhibited by ALA treatment (treat group, 0.59 ± 0.07; *P* = 0.0001) (Figure 5C).

To further verify the effect of ALA on 6-OHDA-induced iron deposit in cells, we exposed PC12 cells to 200 μM of 6-OHDA for 24 h. We found that the PD group showed significantly increased iron contents compared with control groups (control, 0.09 ± 0.03; 6-OHDA, 0.40 ± 0.05; *P* = 0.0001). However, such an increase in iron contents was eliminated dramatically by pretreating the cells with 10 μM of ALA (ALA + 6-OHDA, 0.24 ± 0.04; *P* = 0.0030) (Figure 5D).

### ALA Suppresses 6-OHDA-Induced Upregulation of DMT1 via IRP2 in PC12 Cells and SN of Rats

**FIGURE 6:**
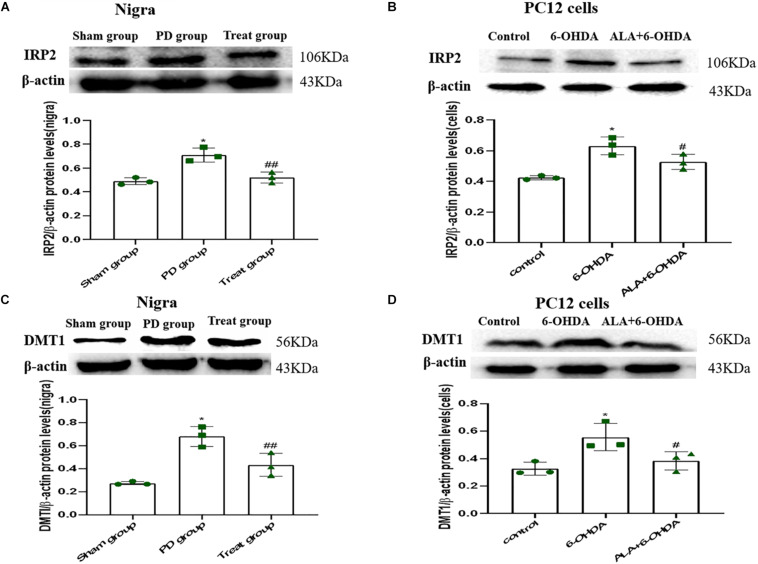
ALA suppresses the 6-OHDA-induced upregulation of DMT1 via IRP2 in the SN of rats and PC12 cells. Rats and cells were treated with 6-OHDA and ALA, as described in section “Materials and methods.” The iron metabolism-related protein levels of IRP2 **(A,B)** and DMT1 **(C,D)** in rat SN and PC12 cells were detected by western blot. Data were expressed as the means ± SD analyzed by one-way ANOVA (*n* = 3). Statistical significance: *P < 0.01 vs. sham group or control; ^##^*P* < 0.01, ^#^*P* < 0.05 vs. PD group or 6-OHDA.

To verify whether the protective mechanism of ALA correlates with iron imbalance induced by 6-OHDA, we investigated the expression of DMT1 and IRP2. As shown in Figures 6A–D, IRP2 and DMT1 were upregulated after 6-OHDA treatment compared with those in the PC12 cells and SN of rats in the control group (Rats IRP2: sham group, 0.49 ± 0.03; PD group, 0.71 ± 0.06; *P* = 0.0010. Cells IRP2: control, 0.42 ± 0.01; 6-OHDA, 0.63 ± 0.06; *P* = 0.0010. Rats DMT1: sham group, 0.27 ± 0.02, PD group, 0.68 ± 0.09; *P* = 0.0010. Cells DMT1: control, 0.33 ± 0.05; 6-OHDA, 0.6356 ± 0.10; *P* = 0.0090). Interestingly, ALA inhibited the upregulation of IRP2 and DMT1 induced by 6-OHDA, indicating that ALA maintained iron homeostasis *in vitro* and *in vivo* (Rats IRP2: treat group, 0.52 ± 0.05; *P* = 0.0020. Cells IRP2: ALA + 6-OHDA; 0.53 ± 0.05; *P* = 0.0290. Rats DMT1: treat group, 0.43 ± 0.10; *P* = 0.0080. Cells DMT1: ALA + 6-OHDA; 0.38 ± 0.07; *P* = 0.0300).

## Discussion

Brain iron accumulation has been implicated in several chronic neurological diseases, such as AD and PD. Iron concentrations are significantly elevated in human parkinsonian SN within DA neurons (Youdim and Riederer, 1993; Shoham and Youdim, 2002; Götz et al., 2004; Gerlach et al., 2006). Iron, an essential nutrient for life, participates in ROS activation to provoke oxidative cell damage (Zhou et al., 2018b). In addition, some studies showed that iron triggered cell death via the ROS pathway, suggesting that iron likely plays a role in ROS signaling (Bogdan et al., 2016). Excess iron participates in the Fenton chemistry, reacting with H_2_O_2_ to produce the most toxic ROS, the hydroxyl radical. In PD, the toxic ROS combined with the depletion of endogenous antioxidants, particularly the levels of SOD and GSH, subsequently increased the levels of induced lipid peroxidation. Increased basal lipid peroxidation induced by oxidative stress is positively correlated with elevated iron levels in the SN of PD (Mash et al., 1991).

6-hydroxydopamine, a neurotoxin that can cause degeneration of DA neurons in animal models and cell apoptosis in PC12 cells, has been widely used in PD models (Glajch et al., 2012; Xu et al., 2001). Recently, increasing evidence suggested that 6-OHDA may selectively cause the death of nigral neurons or dopaminergic neurons by inducing high levels of iron and oxidative stress (Lee et al., 2018; Xu et al., 2018). Thus, inhibiting the elevated iron and oxidative stress induced by 6-OHDA is a potential strategy for protecting neurons.

As a therapeutic agent in the treatment of diabetes mellitus, ALA exerts neuroprotective functions against neurodegenerative diseases in clinical trials and *in vitro*/*vivo* studies, and the protective mechanisms are related to its antioxidant effect (Zhao et al., 2017; Zhou et al., 2018a). In addition to its antioxidant properties, ALA has iron-chelating properties. However, in addition to its antioxidant effect, other potential mechanisms of neuroprotective effects of ALA on PD remain unclear, such as its iron chelation.

Previous studies showed that the neuroprotective effects of ALA are related to its antioxidant properties and iron chelation (Suh et al., 2005; Abdin and Sarhan, 2011; Zaitone et al., 2012; Jalali-Nadoushan and Roghani, 2013). However, the specific mechanism of ALA on PD is still unclear. Based on the iron chelation and antioxidant effects of ALA, the present study investigated the possible role of ALA in a PD model *in vivo* and *in vitro* induced by 6-OHDA. The results suggested that ALA could significantly attenuate the damage of PC12 cells induced by 6-OHDA, promote the survival of DA neurons by inhibiting the decrease in TH expression in the SN of 6-OHDA-induced PD rats, and remarkably ameliorate motor deficits. The results also showed that ALA could significantly suppress the levels of oxidative stress and iron. The results further showed that ALA could attenuate iron accumulation via antagonized 6-OHDA-induced upregulation of IRP2 and DMT1 both *in vivo* and *in vitro*.

Cell injury during the progress of PD is associated with the overproduction of ROS (Gaspar et al., 2015). Numerous studies on postmortem brain tissues of PD patients have suggested that ROS are involved in the degeneration of dopaminergic neurons (Danielson and Andersen, 2008). The endogenous protective antioxidant system of GSH and SOD provides an important defense against oxidative stress (Fridovich, 1995). To further investigate the antioxidant property and neuroprotective mechanism of ALA, we tested the antioxidant SOD and GSH in the SN of rats, and the level of ROS also was tested in PC12 cells. The current results showed that ALA treatment could increase the contents of SOD and GSH in 6-OHDA-treated rats. In addition, ALA pretreatment could inhibit ROS production in 6-OHDA-treated PC12 cells. These results demonstrated that antioxidant activity was essential for ALA to exert its neuroprotective effect on 6-OHDA-induced oxidative damage.

Enhanced iron level was observed in dopaminergic neurons in humans, and iron accumulation has also been shown in the SN of both 6-OHDA- and MPTP-induced animal models (Wang et al., 2007; Hare et al., 2013). Increased iron may induce a vicious cycle of oxidative stress by increasing the levels of free iron (Reif, 1992). In fact, in 6-OHDA-treated rats, increased iron levels and neuronal loss are apparent in as short a time as the SN 1 day following 6-OHDA injection (Wang et al., 2004). Our results were consistent with these studies. 6-OHDA could increase the levels of iron of rat SN. Moreover, 6-OHDA-induced PC12 cells also showed iron accumulation. Interestingly, ALA could significantly inhibit the iron increase in rat SN and PC12 cells.

To explore the mechanism of ALA in inhibiting iron accumulation, we observed some proteins involved in iron homeostasis regulation. *In vitro* and *in vivo* studies showed that the change in DMT1 involved increases iron accumulation (Fridovich, 1995). DMT1 is responsible for iron uptake. IRP1 and IRP2, two subunits of IRPs, can regulate intracellular iron levels and maintain iron homeostasis. IRP1 plays a critical role in the pulmonary and cardiovascular systems, whereas IRP2 functions in the nervous system and erythropoietic homeostasis (Foot et al., 2008). By binding to IREs, IRPs can posttranscriptionally regulate mRNAs that have IREs in the 3′- or 5′-UTRs. IREs are present in the UTRs of DMT1 (IRE), and the expression of DMT1 (IRE) is regulated by the IRP/IRE mechanism, leading to intracellular iron aggregation in PD (Jalali-Nadoushan and Roghani, 2013). A mutation in DMT1 that impairs iron transport protects rodents against parkinsonism-induced neurotoxins MPTP and 6-OHDA (Salazar et al., 2008). Furthermore, DMT1 is highly expressed in the neurons of the SN in PD (Jia et al., 2015). Consistent with previous reports, we found that 6-OHDA upregulated DMTI by upregulating IRP2, which increased iron in rat SN and PC12 cells. However, our results showed that ALA could antagonize the 6-OHDA-induced upregulation of IRP2 and DMT1 in rat SN and PC12 cells. The present study might be a new mechanism of ALA in protecting neurons in PD induced by 6-OHDA.

The maladjustment of iron metabolism, which was mainly caused by the increase in oxidative stress level, leads to the deposition of iron in PD patients. Our analysis of the effects of ALA on iron levels *in vivo* and *in vitro* showed that it indeed inhibited iron accumulation. In addition, ALA treatment could increase the contents of SOD and GSH and could inhibit ROS production in PD models. These results demonstrated that ALA reduces iron deposition, which may be achieved by reducing the levels of oxidative stress and then regulating the disorder of iron metabolism.

Our study showed that ALA attenuated 6-OHDA-induced iron accumulation both *in vivo* and *in vitro* by antagonizing the 6-OHDA-induced upregulation of IRP2 and DMT1. However, the specific mechanisms are unclear. The nuclear factor (erythroid-derived 2)-like 2 (NRF2) transcription factor which can respond to oxidative and electrophilic stress regulates several genes involved in iron metabolism (Kawai et al., 2011). IRP2 and DMT1 may be important to cell iron homeostasis, but the NRF2 factor may be linked to IRP2/DMT1-related iron accumulation. The effects of 6-OHDA-induced neurotoxicity is linked to modulation of the antiaging gene Sirtuin 1 (Sirt 1), which is important to neurodegenerative diseases such as PD (Zou et al., 2016). In addition, ALA is a Sirt 1 activator and actives Sirt 1 (Chen et al., 2012). Hence, ALA may active Sirt 1 by reducing the level of oxidative stress and regulate iron metabolism by the Sirt 1-NRF2-IRP2 pathway and then reduce the iron accumulation of PD models. This may be a new mechanism of ALA in protecting neurons in PD induced by 6-OHDA.

## Conclusion

In summary, ALA displays protective effects against the damage induced by 6-OHDA in PD models, and its neuroprotective mechanism might be associated with improvement of oxidative stress and iron metabolism levels. Increased stress resulting from ROS production is one of the proposed mechanisms for the death of dopaminergic neurons in PD. In addition, iron metabolism dysfunction plays a key role in PD. Hence, ALA has a therapeutic potential for the treatment of PD.

## Data Availability Statement

The datasets analyzed in this article are not publicly available. Requests to access the datasets should be directed to jiaoling5151@sina.com.

## Ethics Statement

All animal experiments in review were carried out in accordance with the National Institutes of Health Guide for the Care and Use of Laboratory Animals and were approved by the Ethical Committee of the Guizhou Medical University.

## Author Contributions

ST and QZ wrote the manuscript. CZ conceived and designed the study. ST, SZ, TC, LX, and LY performed the experiments. LJ and CZ reviewed and edited the manuscript. All authors read and approved the manuscript.

## Conflict of Interest

The authors declare that the research was conducted in the absence of any commercial or financial relationships that could be construed as a potential conflict of interest.
